# Positron Annihilation Lifetime Spectroscopy Insight on Free Volume Conversion of Nanostructured MgAl_2_O_4_ Ceramics

**DOI:** 10.3390/nano11123373

**Published:** 2021-12-13

**Authors:** Halyna Klym, Ivan Karbovnyk, Sergei Piskunov, Anatoli I. Popov

**Affiliations:** 1 Specialized Computer Systems Department, Lviv Polytechnic National University, 12 Bandera Str., 79013 Lviv, Ukraine; ivan_karbovnyck@yahoo.com; 2 Department of Electronics and Computer Technologies, Ivan Franko National University of Lviv, 50 Dragomanov Str., 79005 Lviv, Ukraine; 3 Institute for Solid State Physics, University of Latvia, Kengaraga 8, LV-1063 Riga, Latvia; sergejs.piskunovs@lu.lv

**Keywords:** nanostructured ceramics, positron annihilation, positronium decay, positron trapping, free-volume defects, nanopores

## Abstract

Herein we demonstrate the specifics of using the positron annihilation lifetime spectroscopy (PALS) method for the study of free volume changes in functional ceramic materials. Choosing technological modification of nanostructured MgAl_2_O_4_ spinel as an example, we show that for ceramics with well-developed porosity positron annihilation is revealed through two channels: positron trapping channel and ortho-positronium decay. Positron trapping in free-volume defects is described by the second component of spectra and ortho-positronium decay process by single or multiple components, depending on how well porosity is developed and on the experimental configuration. When using proposed positron annihilation lifetime spectroscopy approaches, three components are the most suitable fit in the case of MgAl_2_O_4_ ceramics. In the analysis of the second component, it is shown that technological modification (increasing sintering temperature) leads to volume shrinking and decreases the number of defect-related voids. This process is also accompanied by the decrease of the size of nanopores (described by the third component), while the overall number of nanopores is not affected. The approach to the analysis of positron annihilation lifetime spectra presented here can be applied to a wide range of functional nanomaterials with pronounced porosity.

## 1. Introduction

Positron annihilation lifetime spectroscopy (PALS) technique is considered as one of the promising alternative methods to analyze free volume and defects in functional and other materials [1,2,3,4,5], including ceramics [6,7,8], glasses [9,10,11], polymers [12,13,14], nanocomposites [15,16,17], etc. There are already several attempts to develop a phenomenological model describing the processes of positron annihilation in metal powders that contain Cu-, W-, Ni- [18], some types of BaTiO_3_ [19,20,21] and SrTiO_3_ perovskites [22,23], nanocrystallite ferrites [24,25], Ni–Cr alloy [26], In_2_O_3_ nanocrystals [27], irradiated W and Fe [28], water diffusivity transition in composites [29,30] and others. Different approaches to the analysis of annihilation spectra as well as decompositions with a different number of components were introduced. It was shown that the main positron annihilation channels in these materials can be related to positron trapping and ortho-positronium (o-Ps) decay. The best results were achieved when using the decomposition involving three independent components [31,32,33]. In the frame of this model, the second component describes capturing of positrons by free volume defects such as vacancy clusters, neutral surfaces of powder particles, or vacancies with a negative charge, particularly those that are close to grain boundaries. The shortest component is related to the annihilation of the defect-free mass with slight mixing with other positron trapping channels and para-positronium (p-Ps). The longest third component corresponds to ortho-positronium (o-Ps) atoms decay [34].

Earlier, we have carried out PALS investigations aimed at exploring free volume changes in Ge-Ga-S(Se) chalcogenide glasses under thermal influences [35], compositional modification (in particular, CsCl addition) and during crystallization processes [36,37,38,39]. Additionally, the investigation of moisture adsorption processes on the free volume changes in MgAl_2_O_4_ ceramics was carried out [25,40,41]. For each of mentioned functional materials, the appropriate models of positron annihilation were suggested, assuming two, three- or four-component decomposition depending on the structural peculiarities. The approach to PALS analysis that allows estimating the influence of additional modifiers on the free volume change was also proposed [38]. However, for most of the mentioned approaches, the initial analysis is often significantly complicated.

The goal of this work is to present a universal approach that can be used to study free volume in functional ceramic materials with pronounced porosity.

The approach is explained in the example of MgAl_2_O_4_ spinel ceramics. MgAl_2_O_4_ ceramics are characterized by developed grain structure, grain boundaries, and pores. Change of the free volume in this material due to technological modification can be considered a model example for a study by means of PALS. A demonstrated approach to the decomposition and the analysis of annihilation spectra of ceramics should allow the application of the technique in the case of other functional nanoporous materials.

## 2. Materials and Methods

MgAl_2_O_4_ ceramics understudy was obtained from initial MgO powders with the specific surface of 10.7 ± 2 m^2^/g and Al_2_O_3_ powders with the specific surface of 12.4 ± 2 m^2^/g, taken in 1:1 molecular ratio. To modify the ceramics, the highest temperatures of isothermal sintering were used (1100 °C, 1200 °C, 1300 °C, and 1400 °C) and sintering durations were set to 2 h, 5 h, and 9 h. In greater detail, the technology of MgAl_2_O_4_ ceramics preparation is described in [41,42].

According to the results of X-ray diffraction [42], ceramics sintered at 1100–1200 °C during 2 h exhibits reflexes of three phases: along with the principal MgAl_2_O_4_ spinel phase, there are MgO (11.25% for the ceramics sintered at 1100 °C and 5.82% for the ceramics sintered at 1200 °C) and Al_2_O_3_ (8.13% for the ceramics sintered at 1100 °C and 6.06% for the ceramics sintered at 1200 °C) phases. Ceramics sintered at 1300 °C and 1400 °C temperatures during 2 h show only reflexes of the single additional phase MgO in the amount of 3.5 and 1.5%, respectively. Similar values were observed for ceramics sintered during 5 and 9 h.

Evolution of free volume in MgAl_2_O_4_ ceramics was experimentally studied by PALS method using ORTEC spectrometer (with conventional fast-fast coincidence system of 270 ps resolution, full width at half maximum FWHM of a single Gaussian, determined by 60Co isotope measuring) at the temperature *T* = 22 °C and relative humidity *RH* = 35% [43,44]. For each pair of samples under study, three spectra of PALS were collected. The difference between these spectra was in the number of ordinary annihilation events that were in the range of 800,000 to 1,200,000. Each spectrum was subjected to multiple processing by the LT program due to small changes in the number of final channels, annihilation background, and the time shift of the spectrum. The best results were selected based on the least-squares fit between experimental points and theoretical curve [45]:

(1) $$FIT=\frac{\sum_{k=1}^{N}{\left(\frac{T_{k}-E_{k}}{\sqrt{E_{k}}}\right)}^{2}}{N-m}\approx \frac{1}{N}\sum_{k=1}^{N}{\left(\frac{T_{k}-E_{k}}{\sqrt{E_{k}}}\right)}^{2},$$

where *N* is the number of channels (or the number of experimental points), *E_k_*–measured counts in the *k*-th channel, *T_k_*–theoretical counts in the *k*-th channel, $\sqrt{E_{k}}$–mean square deviation of counts in the *k*-th channel and m is the number of fitting parameters. The value *T_k_* is selected automatically in the LT program depending on the selected model, which best describes the theoretical curve.

As a result, several data groups having a different number of experimental points *N* were formed within the selected fitting procedure. Only results with FIT values close to 1 (optimal deviation was in the range from 0.95 to 1.2) were considered as optimal ones within the chosen model. In the next step, these values and determined PALS characteristics were controlled depending on the annihilation background and time shift of the PALS spectrum, the results show only minor changes selected by us. It should be noted that the source correction and spectrometer resolution function remained unchanged in the above algorithm.

Since low statistic measurement mode was exploited, spectra were decomposed into three components by means of LT software (version 10.2.2) [46]. Best results selection was done in steps. First, results with FIT exceeding 1.2 were dropped. Second, groups with close FIT values in increasing order were formed and results with various values of lifetimes within one group were ignored. Third, for preliminary assessment, average positron lifetimes were calculated for each group. Next, FIT values, lifetimes, and intensities were averaged for each group. Ultimately, the best results with minimum FIT values were chosen and used for further analysis.

By processing the PALS spectra by the LT program, it is possible to obtain the values of the fitting parameters, i.e., lifetimes and intensities with an accuracy of ±0.001 ns and ±0.1%, respectively. However, given that the accuracy of lifetime measurements using the ORTEC spectrometer, in this case, is ±0.01 ns, the lifetimes obtained in the process of mathematical adjustment and the intensities of the respective components were rounded to 0.01 ns and 0.01 a.u. (or 1%), respectively.

## 3. Results and Discussion

As shown in [41,44], the best results of PALS spectra processing in LT software for MgAl_2_O_4_ ceramics can be achieved with the three-component fitting procedure (in the case of low statistic measurement mode). Therefore, this approach was applied for the analysis of extended positron-trapping defects and nanopores in technologically modified ceramics sintered at 1100–1400 °C during 2, 5, and 9 h.

**Figure 1:**
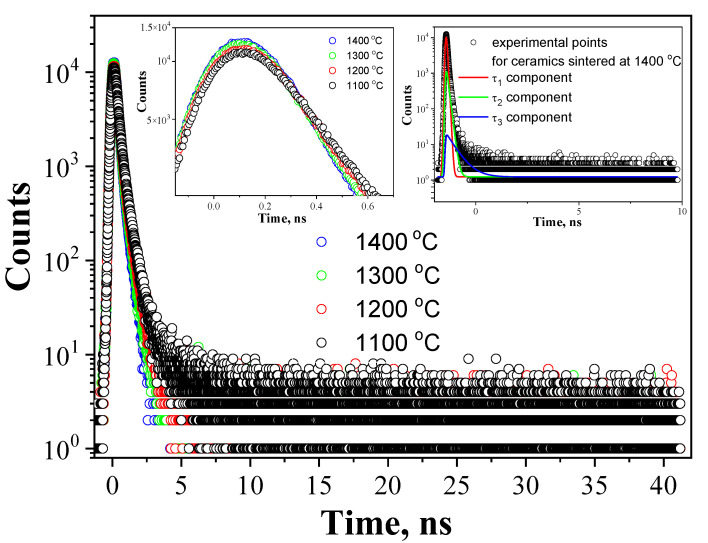
PALS spectra for MgAl_2_O_4_ ceramics sintered at 1100–1400 °C for 2 h with three components decomposition curves for ceramics sintered at 1400 °C.

PALS spectra for MgAl_2_O_4_ ceramics sintered at 1100–1400 °C during 2 h with three components decomposition for ceramics sintered at 1400 °C are shown in Figure 1. 

Typical spectra for ceramics are characterized by a narrow peak and long smooth decay region, where counts are decreased with time. Mathematical three-component decomposition is represented by the sum of exponential decay functions with different powers inversely proportional to the positron lifetimes *τ*_1_, *τ*_2_, and *τ*_3_. The areas under each curve are proportional to intensities *I*_1_, *I*_2_, and *I*_3_.

Besides the main fitting parameters (positron lifetimes *τ*_1_, *τ*_2_ and *τ*_3_ and intensities *I*_1_, *I*_2_, and *I*_3_) that are acquired directly in LT software, the average positron lifetime *τ*_av_ that reflects the properties of the prevailing defect environment in the material was calculated using two-state positron trapping model [45]:

(2) $$\tau_{av.}=\frac{\tau_{1}I_{1}+\tau_{2}I_{2}}{I_{1}+I_{2}}.\;$$

We have also estimated the lifetime *τ*_b_ related to the annihilation of positrons in the defect-free region:

(3) $$\tau_{b}=\frac{I_{1}+I_{2}}{\frac{I_{1}}{\tau_{1}}+\frac{I_{2}}{\tau_{2}}}.\;$$

Trapping rate *κ*_d_ at which positrons are captured by defects was calculated as follows:

(4) $$\kappa_{d}=\frac{I_{2}}{I_{1}}\left(\frac{1}{\tau_{b}}-\frac{1}{\tau_{2}}\right).$$

For spinel ceramics, the difference *τ*_2_ − *τ*_b_ is treated as an average size of the defect region where positrons are trapped, while *τ*_2_/*τ*_b_ ratio is looked at as the parameter that reflects the nature of volume defects [44]. 

In [44,46,47] it was shown that for functional ceramic materials two PALS channels are enabled: “free” positron trapping (the component with lifetime *τ*_2_) and o-Ps decaying (component with lifetime *τ*_3_). Within the two-state positron trapping model, the first component with lifetime *τ*_1_ and intensity *I*_1_ includes free annihilation, p-Ps decay and is related also to the positrons’ bulk lifetimes *τ*_b_ in the samples. For some materials (for example, chalcogenide glasses [35,36,37,38,39]) this component has no physical meaning. In the frame of the proposed unified model [44], in MgAl_2_O_4_ ceramics, the first component with parameters *τ*_1_ and *I*_1_ reflects mainly microstructural specifics of spinel ceramics with characteristic octahedral and tetrahedral vacant cation sites along with a contribution from the annihilation of p-Ps atoms which is not considered in the further analysis. The lifetime *τ*_2_ is related to the size of free-volume defects (voids) near grain boundaries with additional phases and *I*_2_ intensity reflects their amount. The third component (*τ*_3_, *I*_3_) originates from the annihilation of o-Ps atoms in intrinsic nanopores of MgAl_2_O_4_ ceramics. 

**Figure 2:**
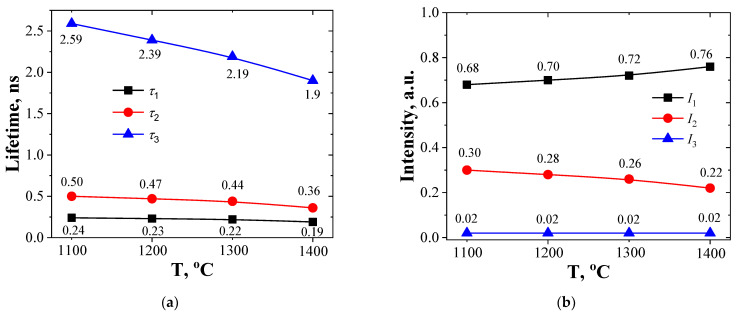
Dependencies of lifetimes (**a**) and intensities (**b**) of the PALS spectrum on the ceramics sintering temperature.

As can be seen from Figure 2, for nanostructured MgAl_2_O_4_ ceramics obtained at 1100–1400 °C during 2 h, lifetime *τ*_1_ of the first short component is decreasing slightly with increasing *T*_s_, whereas intensity *I*_1_ is growing. Such changes speak in favor of ceramics quality increasing towards higher perfection level when using higher sintering temperatures.

Lifetime of the second component *τ*_2_ is related to the positron trapping in defect-related sites. As known from X-ray diffraction, MgAl_2_O_4_ ceramics contain different amounts of MgO/Al_2_O_3_ phases [42]. These amounts decrease with increasing *T*_s_. As confirmed by scanning electron microscopy studies [42], additional phases are irregularly distributed across the ceramics volume and are mainly localized near grain boundaries. Separated MgO and Al_2_O_3_ phases play the role of specific positron trapping centers in the ceramics free volume. Since ceramics obtained at lower temperatures include larger amounts of additional phases, positron trapping in such samples should be more pronounced.

From Figure 2 it also follows that the lifetime and intensity of the second component (*τ*_2_ and *I*_2_, respectively) are essentially decreased with increasing *T*_s_. Lifetime is in correlation with the size of free-volume extended defects (positron trapping center) near grain boundaries and intensity corresponds to the amount of these extended defects. Therefore, with increasing sintering temperature at a fixed 2 h duration one observes the decrease of the free volume where positrons are trapped and the decrease in the number of defects.

**Figure 3:**
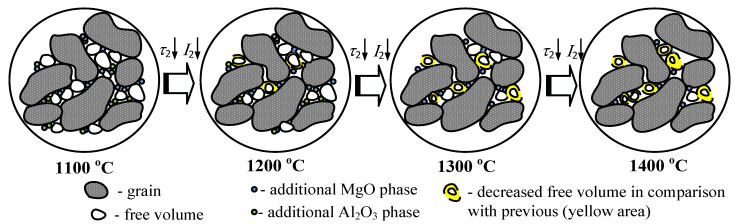
Schematic depiction of the evolution of internal free volume defects at grain boundaries in MgAl_2_O_4_ ceramics.

Schematically the evolution of the free volume at grain boundaries in the process of the discussed technological modification of MgAl_2_O_4_ ceramics is shown in Figure 3.

**Figure 4:**
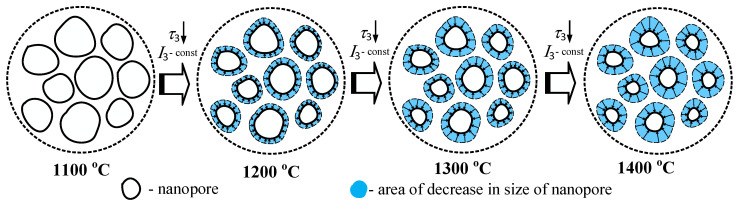
Diagram explaining the evolution of nanopores near grain boundaries in MgAl_2_O_4_ ceramics sintered at 1100–1400 °C during 2 h.

The third long component of the PALS spectrum (the second channel of positron annihilation with lifetime *τ*_3_ and intensity *I*_3_) is connected to the decay of o-Ps atoms in nanopores and also to the “pick-off” annihilation process [45,48]. As summarized in Figure 2, lifetime *τ*_3_ decreases from 2.59 ns down to 1.9 ns when *T*_s_ increases from 1100 °C to 1400 °C. At the same time, intensity *I*_3_ remains unchanged and equals 0.02 a.u. Such behavior indicates the decrease of nanopores size and an unchanged overall number of nanopores. Altogether this means more efficient sintering at higher temperatures. The diagram in Figure 4 further explains the evolution of nanopores near grain boundaries in ceramics.

Values of other parameters related to the positron trapping on defects (*τ*_av_, *τ*_b_, *κ*_d_) slightly decrease with increasing *T*_s_, which is in fair agreement with the number of additional phases existing in the ceramics understudy near grain boundaries (see Table 1). Even though extended positron trapping defects have an almost identical structural and chemical origin, the value of (*τ*_2_ − *τ*_b_) is larger in ceramics sintered at lower temperatures. Free-volume geometry (*τ*_2_/*τ*_b_ ratio) remains at the level of 1.7. Probably, in these ceramics, the same positron trapping defect center (extended defects near grain boundaries) with a characteristic size of about one-two atomic vacancies prevails, and positron trapping locations have the same nature.

Next technological structural modification of MgAl_2_O_4_ ceramics was done by increasing sintering duration up to 5 and 9 h while keeping the temperature fixed at 1300 °C and 1400 °C. Results are summarized in Table 2.

**Figure 5:**
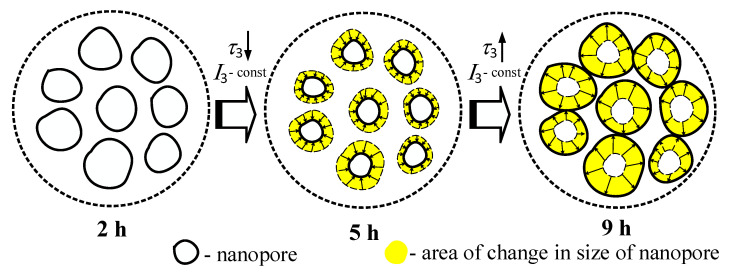
Scheme of nanopores evolution near grain boundaries in MgAl_2_O_4_ ceramics sintered at 1300 °C during 2, 5, and 9 h.

As can be concluded from the data of Table 2, lifetimes of the first and the second components (*τ*_1_ and *τ*_2_, respectively) and intensity *I*_2_ decrease with increasing the sintering duration from 2 h to 5 h and 9 h, while intensity *I*_1_ increases following the increase of principle MgAl_2_O_4_ spinel ceramics phase amount. Decrease of *I*_2_ from 0.32 down to 0.25 a.u. with increasing sintering duration from 2 to 9 h in the ceramics that were sintered at 1300 °C is related to the decrease in the amount of free volume defect-related positron trapping centers created due to separated additional phases near grain boundaries. Similar changes were observed in the case of increased ceramics sintering temperature (see Figure 2). In the ceramics sintered at 1400 °C during 5 and 9 h, lifetimes and intensities of the first and the second components are not changed. This is because the amount of an additional MgO phase is almost the same [42]. The lifetime of the third component at the constant intensity *I*_3_ = 0.01 in the ceramics sintered at 1300 °C increases when the sintering duration is 9 h. This indicates the increase of the size of the nanopore near grain boundaries which might have a negative effect on the functional properties of these ceramics. A schematic depiction of the process is presented in Figure 5.

In the ceramics sintered at 1400 °C, lifetime *τ*_3_ is decreased and close to the value that reflects the “pick-off” annihilation of o-Ps in water. It is likely that in such ceramics additional moisture adsorption is possible. Positron trapping parameters as expected to be not significantly different for various ceramics sintering durations (2, 5, and 9 h) at 1300 °C and 1400 °C temperatures.

Additionally, using the lifetime of the third component we can calculate nanopores radii assuming spherical approximation and using the Tao-Eldrup model [49,50,51,52]:

(5) $$\tau_{o-Ps}={\left[2\left(1-\frac{R}{R+\Delta R}+\frac{1}{2\pi }sin\left(\frac{2\pi R}{R+\Delta R}\right)\right)+0.007\right]}^{-1},\;$$

where Δ*R* is empirically obtained parameter (Δ*R* ≈ 0.1656 nm) describing effective electron layer thickness related to the “pick-off” annihilation of o-Ps in an empty space.

**Figure 6:**
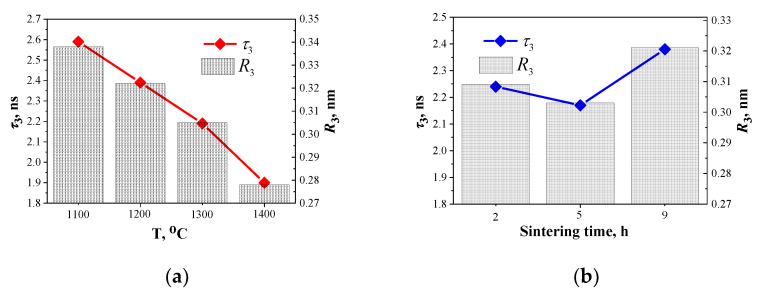
Dependencies of lifetime *τ*_3_ and nanopores radius *R*_3_ calculated according to Tao-Eldrup model for MgAl_2_O_4_ ceramics obtained at 1100–1400 °C and sintering duration of 2 h (**a**) and at 1300 °C and sintering duration 2, 5, and 9 h (**b**).

Calculation results are provided in Table 1 and Table 2 as well as in Figure 6. Nanopores radii calculated based on lifetimes values of the third component for MgAl_2_O_4_ ceramics vary within the range of 0.28–0.34 nm. This can serve as a validation of the fact that the PALS method can also be used to determine nanovoids size in functional materials.

## 4. Conclusions

Positron annihilation processes in functional ceramic materials with pronounced porosity (on the example of technologically modified nanostructured MgAl_2_O_4_ ceramics) are described within a two-channel model: positron trapping and o-Ps atoms decay channels. In low statistic measurement mode, better results in PALS analysis are achieved when using three-component decomposition. The first component reflects the main microstructural features of spinel ceramics with tetrahedral and octahedral vacancies, the second one corresponds to the extended free-volume defects (positron trapping sites) localized near grain boundaries and the third component describes the annihilation of o-Ps atoms in nanopores.

Technological conditions of MgAl_2_O_4_ ceramics preparation (maximum temperature and duration of sintering) are the factors that define the annihilation spectra of positron lifetimes. Positron lifetimes of the first and the second components and the intensity of the second component *I*_2_ obtained from investigated PALS spectra decrease, while the intensity of the first component *I*_1_ increases for more perfect ceramics structure with *I*_3_ remaining unchanged. This is evidence of better sintering of ceramic grains accompanied by decreasing defect-related free volume at grain boundaries and nanopores size with an overall amount of nanopores basically unaffected.

Results obtained by PALS can serve as a research background for the development of independent methods of diagnosing nanosized free volumes in ceramic materials, including neutron and heavy-ion irradiated MgAl_2_O_4_ spinels [53,54,55], Si_3_N_4_ [56], Ge_3_N_4_ [57], and AlN [58,59,60] (which are especially promising as diagnostic materials for EUROfusion applications) and also facilitate understanding of porosity, development, and transformation of pores in electrochemical and other devices for energy conversion [61,62,63,64,65,66,67,68].

## Figures and Tables

**Table 1:** Positron trapping parameters and nanopores radii for MgAl_2_O_4_ ceramics sintered at *T*_s_ = 1100–1400 °C during 2 h.

*T*_s_, °C	*τ*_av_, ns	*τ*_b_, ns	*κ*_d_, ns^−1^	*τ*_2_ − *τ*_b_, ns	*τ*_2_/*τ*_b_	*R*_3_, nm
1100	0.32	0.28	0.65	0.21	1.72	0.338
1200	0.30	0.27	0.63	0.20	1.74	0.322
1300	0.27	0.25	0.62	0.19	1.74	0.305
1400	0.24	0.21	0.56	0.15	1.69	0.278

**Table 2:** PALS characteristics for MgAl_2_O_4_ ceramics obtained at *T*_s_ = 1300–1400 °C during 2, 5, and 9 h.

*T*_s_, °C/h	*τ*_1_, ns	*I*_1_, a.u.	*τ*_2_, ns	*I*_2_, a.u.	*τ*_3_, ns	*I*_3_, a.u.	*τ*_av_, ns	*τ*_b_, ns	*κ*_d_, ns^−1^	*τ*_2_ − *τ*_b_, ns	*τ*_2_/*τ*_b_	*R*_3_, nm
1300/2	0.17	0.67	0.40	0.32	2.24	0.01	0.24	0.21	1.0	0.19	1.9	0.309
1300/5	0.16	0.71	0.38	0.28	2.17	0.01	0.22	0.19	1.0	0.19	2.0	0.303
1300/9	0.15	0.74	0.37	0.25	2.38	0.01	0.21	0.18	1.0	0.19	2.1	0.321
1400/2	0.16	0.78	0.38	0.21	2.18	0.01	0.20	0.18	0.9	0.20	2.1	0.304
1400/5	0.15	0.77	0.37	0.22	2.17	0.01	0.20	0.17	0.9	0.20	2.2	0.303
1400/9	0.15	0.77	0.37	0.22	1.83	0.01	0.20	0.17	0.9	0.20	2.2	0.271

## Data Availability

The data presented in this study are available on request from the corresponding author.
